# HashSeq: a Simple, Scalable, and Conservative *De Novo* Variant Caller for 16S rRNA Gene Data Sets

**DOI:** 10.1128/mSystems.00697-21

**Published:** 2021-11-09

**Authors:** Farnaz Fouladi, Jacqueline B. Young, Anthony A. Fodor

**Affiliations:** a Department of Bioinformatics and Genomics, University of North Carolina at Charlotte, Charlotte, North Carolina, USA; University of Copenhagen

**Keywords:** 16S rRNA gene sequence variant, microbiome, sequence variant, sequencing error

## Abstract

16S rRNA gene sequencing is a common and cost-effective technique for characterization of microbial communities. Recent bioinformatics methods enable high-resolution detection of sequence variants of only one nucleotide difference. In this study, we utilized a very fast HashMap-based approach to detect sequence variants in six publicly available 16S rRNA gene data sets. We then use the normal distribution combined with locally estimated scatterplot smoothing (LOESS) regression to estimate background error rates as a function of sequencing depth for individual clusters of sequences. This method is computationally efficient and produces inference that yields sets of variants that are conservative and well supported by reference databases. We argue that this approach to inference is fast, simple, and scalable to large data sets and provides a high-resolution set of sequence variants which are less likely to be the result of sequencing error.

**IMPORTANCE** Recent bioinformatics development has enabled the detection of sequence variants with a high resolution of only one single-nucleotide difference in 16S rRNA gene sequence data. Despite this progress, there are several limitations that can be associated with variant calling pipelines, such as producing a large number of low-abundance sequence variants which need to be filtered out with arbitrary thresholds in downstream analyses or having a slow runtime. In this report, we introduce a fast and scalable algorithm which infers sequence variants based on the estimation of a normally distributed background error as a function of sequencing depth. Our pipeline has attractive performance characteristics, can be used independently or in parallel with other variant callers, and provides explicit *P* values for each variant evaluating the hypothesis that a variant is caused by sequencing error.

## INTRODUCTION

Amplicon sequencing is a popular and cost-effective method for investigating microbial communities. A challenging step in using amplicon sequencing to identify members of microbial communities is to infer true sequences from artifacts. Sequence errors commonly occur during both PCR amplification and DNA sequencing. These errors include single nucleotide substitutions and gap errors due to mismatching bases and polymerase slippage, respectively (1). For many years, standard practice was to lump sequences with 97% identity together into operational taxonomic units (OTUs) in order to reduce noise and cluster closely related taxa (2–4). However, recently developed bioinformatic tools attempt to infer true biological sequences at 100% identity by estimating the error profile and correcting point errors in sequences through denoising processes (1, 5, 6). These pipelines rely on different assumptions and implement various statistical models. For example, DADA2 models error rate as a function of quality scores for each possible nucleotide transition and then these error rates are used in a Poisson-based model to infer true sequences from sequence errors (5). Deblur compares sequence-to-sequence hamming distances to an upper-bound error model combined with a greedy algorithm (6). Unoise2 uses two parameters that are preset values and are used for filtering low-abundance sequencing and clustering of sequences based on their abundances (1). All of these algorithms provide a higher resolution of taxonomic composition of a microbial community than the traditional OTU picking approach.

Despite the important progress that they represent, these algorithms all have some limitations. Deblur depends on construction of a multiple-sequence alignment, which means that it does not scale to an entire data set but works instead on each sample individually. This leads to the possibility of dependencies on the sequencing depth of each sample where variants might be called as real or artifactual differently in different samples depending on the properties of individual samples. Since Deblur sorts the abundance of sequences in each sample individually, it is also possible that the relative abundance of each variant within each sample can impact overall variant calling in complex ways. Deblur also has a number of free parameters and it is not immediately obvious how to optimize these parameters for new sequence data sets that might have different properties from the Illumina MiSeq and HiSeq training sets that were used for setting Deblur’s default values. Unoise2 is not freely available and also requires user setting of parameters for which optimal values may not be entirely clear. As we will show, DADA2 can sometimes in practice yield larger numbers of sequence variants than can be considered biologically reasonable and often requires additional filtration of low-abundance variants. Since DADA2 uses the Poisson distribution, it assumes that processes that control errors have similar rates for high- and low-abundance variants. These sorts of assumptions can be problematic in genomics. For example, in RNA sequencing (RNA-seq) analysis, it has long been understood that the relationship between mean and variance is dependent on sequencing depth (7).

Here, we present HashSeq, a very simple and fast algorithm for inferring sequence variants. We demonstrate that with enough sequence depth, every possible unique one-mismatch variant for a sequence will be observed. We propose that the inference of true variants can therefore be determined relative to this background probability of observing one-mismatch variants, which can be approximated with a two-parameter normal distribution. We applied this method to six publicly available data sets and show that this simple approach is fast and scales well even to large data sets. Our approach provides a conservative set of variant calls that is well supported by a reference database and behaves almost identically to DADA2 calls in supervised classification.

## RESULTS

### With sufficient sequencing depth, all one-mismatch “child” variants for a “parent” sequence are likely to be observed, and this is well modeled by a simple Poisson process.

**FIG 1:**
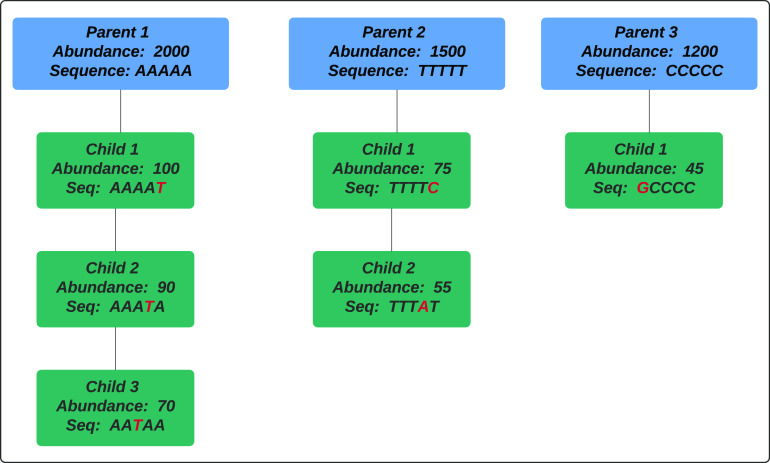
Cluster formation of parents and their one-mismatch children in the HashSeq algorithm. In this clustering strategy, sequence variants are sorted according to their abundances. Starting with the most abundant sequence variant, considered the first parent sequence, clusters are formed by adding all the one-mismatch variants (one-mismatch children) to each cluster.

**FIG 2:**
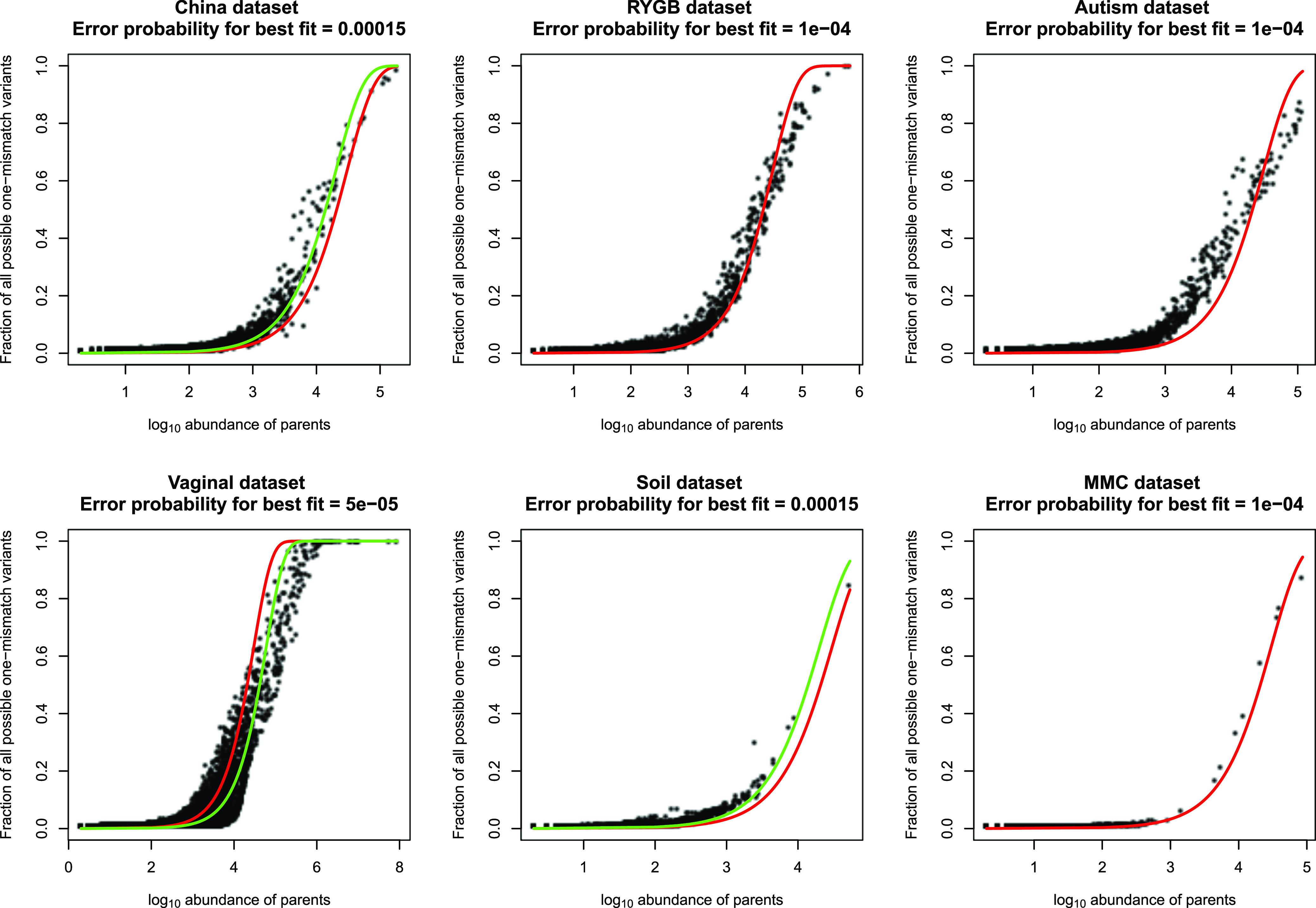
The presence or absence of unique one-mismatch variants can be well modeled with a simple one-parameter Poisson distribution with an almost constant error rate across six independent 16S rRNA gene Illumina data sets. Plots show the relationship between the abundance of parent sequences on the log_10_ scale and the fraction of all possible unique one-mismatch variants for the parent sequences. These data are well modeled by a simple one-parameter Poisson distribution. The red line corresponds to an error rate *P* of 10^−4^. The China, vaginal, and soil data sets were best modeled using slightly different error rates for each data set (green lines, China and soil *P = *1.5 × 10^−4^ and soil *P = *5 × 10^−5^, respectively).

We used a HashMap data structure, which identifies every unique sequence in linear time proportion to the total number of sequences, to identify all sequence variants in six publicly available Illumina data sets. Sequences from these projects were obtained from three fecal microbiota data sets (the China, autism, and Roux-en-Y gastric bypass [RYGB] data sets), one vaginal microbiota data set, and one soil microbiota data set as well as one microbial mock community (MMC; see Materials and Methods). This method of sequence variant detection is very fast (less than 1 h even for the largest data set, with 416,450,026 sequences), but it results in a large number of sequence variants ranging from 6,166 for the smallest data set (mock community) to 814,494 for the largest data set (vaginal data set). The majority of these variants are presumably sequencing errors or other artifacts. In order to detect sequence errors, we clustered sequences that had only one nucleotide difference (Fig. 1). Under this approach, sequence variants were sorted according to their abundances. Starting with the most abundant sequence variant, considered the first “parent sequence,” clusters were formed by adding all the one-mismatch variants to each cluster (one-mismatch “children”). This resulted in 2,002 clusters of parents plus children (when present) for the smallest data set (MMC data set) and 387,903 clusters for the largest data set (vaginal data set). We assessed the relationship between the abundance of parents and the number of one-mismatch children present in each cluster. Figure 2 shows the fraction of all possible one-mismatch children as a function of the abundance of each parent sequence for six data sets. When the abundance of a parent sequence is high enough, almost all possible unique one-mismatch children for that parent sequence can be observed (Fig. 2). For example, the read lengths for both the China and vaginal data sets are 250 bp; therefore, there are 750 possible one-nucleotide differences for a parent sequence in these data sets. For these data sets, the most abundant parent sequences have more than 10,000 reads and almost all of the possible child variants were observed (the rightmost points in Fig. 2). For the least abundant parent sequences (the leftmost points in Fig. 2), almost no one-mismatch variants were observed.

Interestingly, these data were surprisingly well fit with a simple Poisson distribution with a single parameter across all data sets. The single parameter is the probability (*P*) that a single nucleotide will be different between two sequence variants (see Materials and Methods). Even though this model does not contain any information about different error rates for different nucleotides, or any information about the biology of any of these diverse ecosystems, all the data sets were reasonably well fit, with a *P *value of 10^−4^ (Fig. 2, red curves), although for some data sets a slightly better fit could be obtained with a slightly different value for *P* (Fig. 2, green curves). The consistency of this fit across data sets is perhaps surprising given that not all the data sets used the same primers for PCR amplification as well as the wide biological variability of these samples extending from the vaginal to the gut and soil microbiomes. This analysis suggests that a common baseline error rate exists across multiple Illumina data sets and that the probability of seeing a one-mismatch variant is well modeled as a simple function of the abundance of the parent sequence. Our results demonstrate that with enough sequencing depth, every possible one-mismatch child is likely to be observed for all variants and in the absence of any other information, it is possible to predict the likelihood of seeing a unique child variant given only the abundance of the parent.

### The background Poisson distribution underestimates the true abundance of one-mismatch child variants, while a normal distribution-based model provides a better fit.

**FIG 3:**
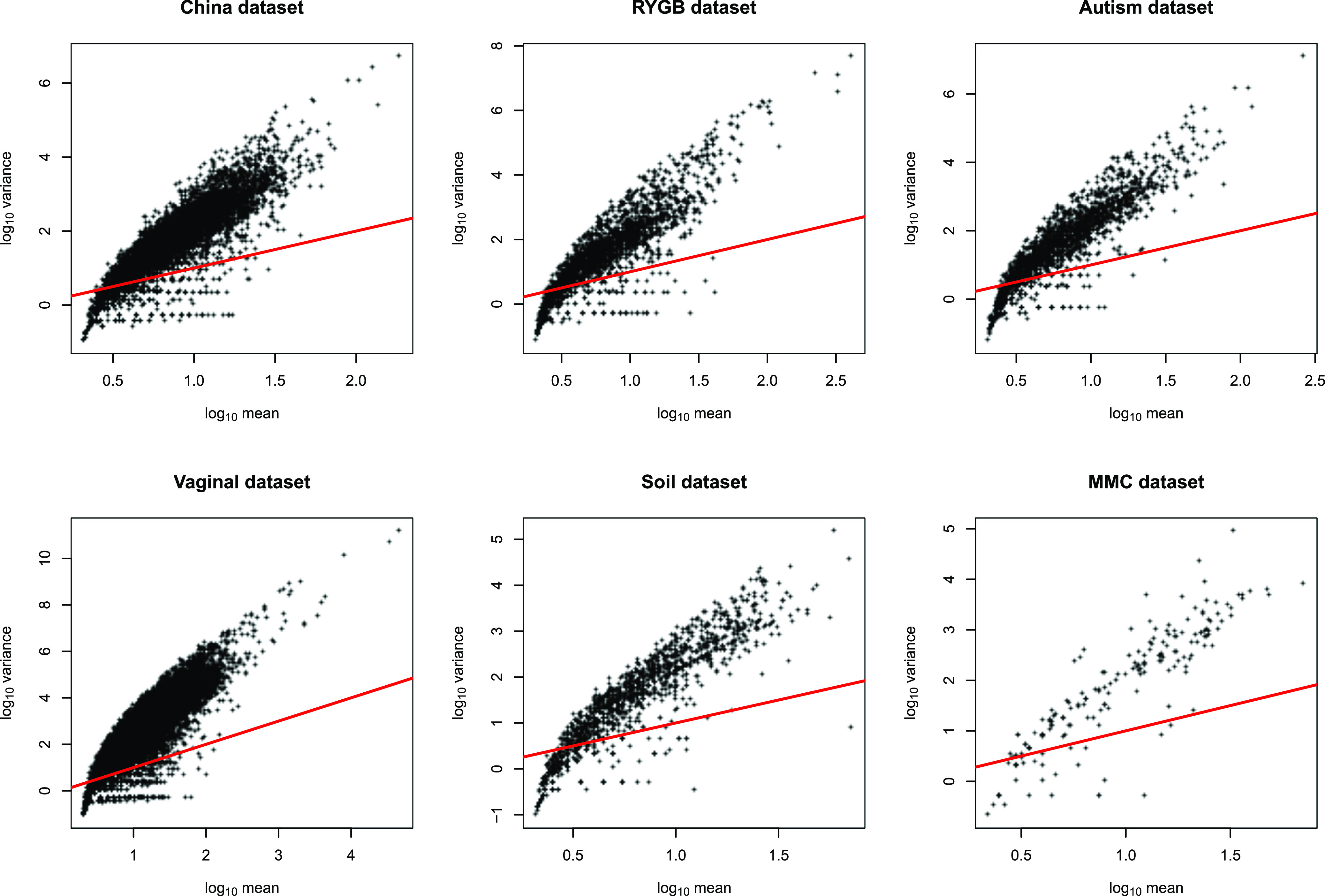
The variance of the one-mismatch child abundance in each cluster is not equal to their mean abundance. Plots show the relationship between the variance and mean abundance of one-mismatch children from each parent cluster across six different 16S rRNA gene data sets. The red line represents the Poisson assumption of equal mean and variance.

Since we have demonstrated that one-mismatch variants accumulate as a simple function of sequencing depth, the challenge for all algorithms in finding sequence variants is to discriminate true variants from the many stochastically produced artifactual variants. One possible approach to this problem might be to use the estimated error rate derived from the presence or absence of one-mismatch variants (as described in the previous section) to predict the background abundance of sequence errors and consider variants “true” only if the abundance of sequence variants is significantly enhanced over the expected background noise. However, when we tried to use this background error rate as a threshold for determining true variants from artifacts using the Poisson test (see Materials and Methods), we rejected the null hypothesis that the sequence variant was due to random sequencing error for more than 83% of one-mismatch children even after correcting for multiple-hypothesis testing (see Fig. S1 in the supplemental material). This suggests that the distribution of child abundance does not follow the Poisson distribution. Indeed, the Poisson distribution assumes that the mean equals the variance, and clearly this assumption does not hold, as the variance of child abundance shows clear overdispersion, that it is larger than the mean of child abundance for most parent sequence variants across data sets (Fig. 3). As a result, the Poisson distribution underestimates the true variance and is therefore anticonservative and calls nearly all one-mismatch variants as true variants.

10.1128/mSystems.00697-21.1 FIG S1 The simple one-parameter Poisson model underestimates the abundance of sequence errors. Plots show the relationship between the abundance of parent sequences and the abundance of one-mismatch child sequences on the log_10_ scale. The Poisson test with error rates estimated from the Poisson model (the best fit for each data set; green lines in Fig. 1 in the main text) was used to test the null hypothesis that a one-mismatch child can be explained by sequencing error. Red and black dots indicate significant and insignificant *P* values, respectively, at a false-discovery rate (FDR) of 5%. Download FIG S1, PDF file, 0.04 MB. Copyright © 2021 Fouladi et al. 2021 Fouladi et al. https://creativecommons.org/licenses/by/4.0/ This content is distributed under the terms of the Creative Commons Attribution 4.0 International license.

**FIG 4:**
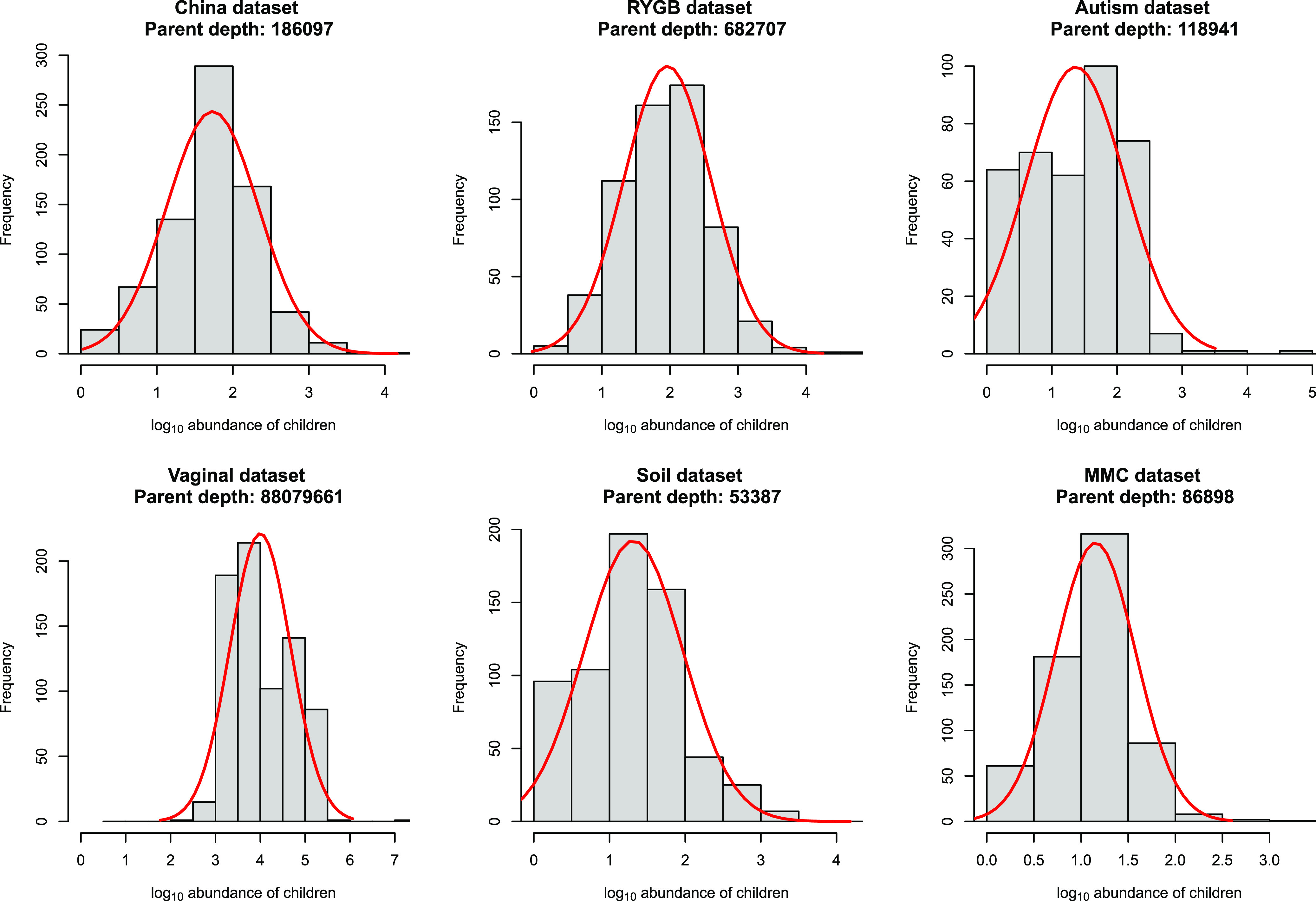
The abundance of one-mismatch children within a cluster is approximately normal on a log_10_ scale. Histograms show the distribution of abundance of one-mismatch children for the most abundant parent on a log_10_ scale across the six different 16S rRNA gene data sets.

**FIG 5:**
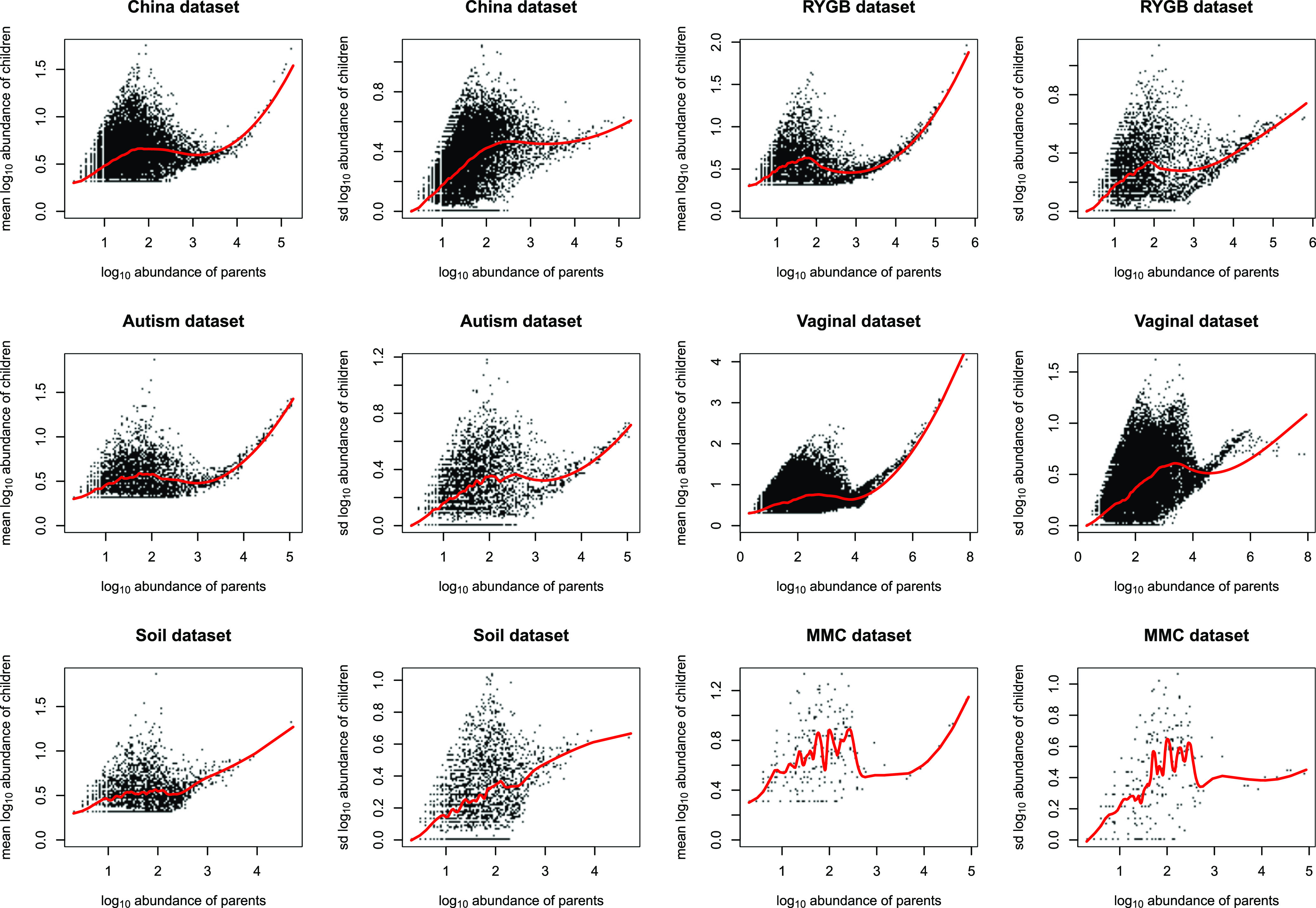
Mean and standard deviation of one-mismatch children in each cluster is a smooth function of their parent abundance on a log_10_ scale for the most abundant parent sequences. Plots show the relationship between the mean and standard deviation of one-mismatch child abundance in each cluster and their parent abundance on a log_10_ scale. The mean and standard deviation of abundance of one-mismatch children for each cluster can be well fit by a smooth LOESS function of the parent abundance especially for high-abundance parents (>1,000 reads) across six different 16S rRNA gene data sets (red line).

As we observed that the Poisson distribution appears to be extremely anticonservative, we next examined whether the distribution of one-mismatch children could be better explained by a normal distribution since it is more flexible in terms of the relationship between the mean and variance than the Poisson distribution. For this, the abundances of children were log_10_ transformed and the distributions of log_10_-transformed abundances of children were plotted for each parent sequence. The histograms of child abundances (shown for the most abundant parent for each data set in Fig. 4) suggest that the distribution of one-mismatch children approximates a normal distribution. Interestingly, we observed that the mean abundance of children for each cluster can be well fit by a locally estimated scatterplot smoothing (LOESS) function of the parent abundance, especially for high-abundance parents (>1,000 reads) across all the six data sets (Fig. 5). The smooth relationship between mean and standard deviation (SD) and sequencing depth across the six data sets suggests that there is a general error rate across all variants that is dependent on sequencing depth but not dependent on the biology of each particular parent sequence. This further suggests that the LOESS fit may represent a good model for general inference. However, when parents have a lower abundance, generally below 1,000 reads across all samples, a smaller number of one-mismatch variants are present (Fig. 1), and therefore, variance in the mean abundance of children significantly increases (Fig. 5), presumably due to sparsity effects.

### Normal-based inference of one-mismatch children is fast and conservative and produces results comparable to those of DADA2 in supervised classification analyses.

The above results suggest that we can assume that the background distribution of child variants is reasonably normally distributed and is well fit for sequences with abundance >∼1,000 reads by a simple localized regression (LOESS). In this section, we explore an inference scheme in which the background mean and standard deviation are the higher of the mean and the standard deviation found for each parent (black dots in Fig. 5) or the LOESS regression of the mean and standard deviation (red lines in Fig. 5). In this scheme, we use these estimates of mean and standard deviation as our background null hypothesis that the abundance of the one-mismatch child variant is a sequence error and can therefore be explained by the background level of sequencing error of the parent. From these background mean and standard deviation, we generate a one-sided *P* value (using “pnorm” in R) for rejecting the null hypothesis. A small *P* value for this null hypothesis indicates that a child variant has an abundance level above this expected background for its parent (see Materials and Methods).

**FIG 6:**
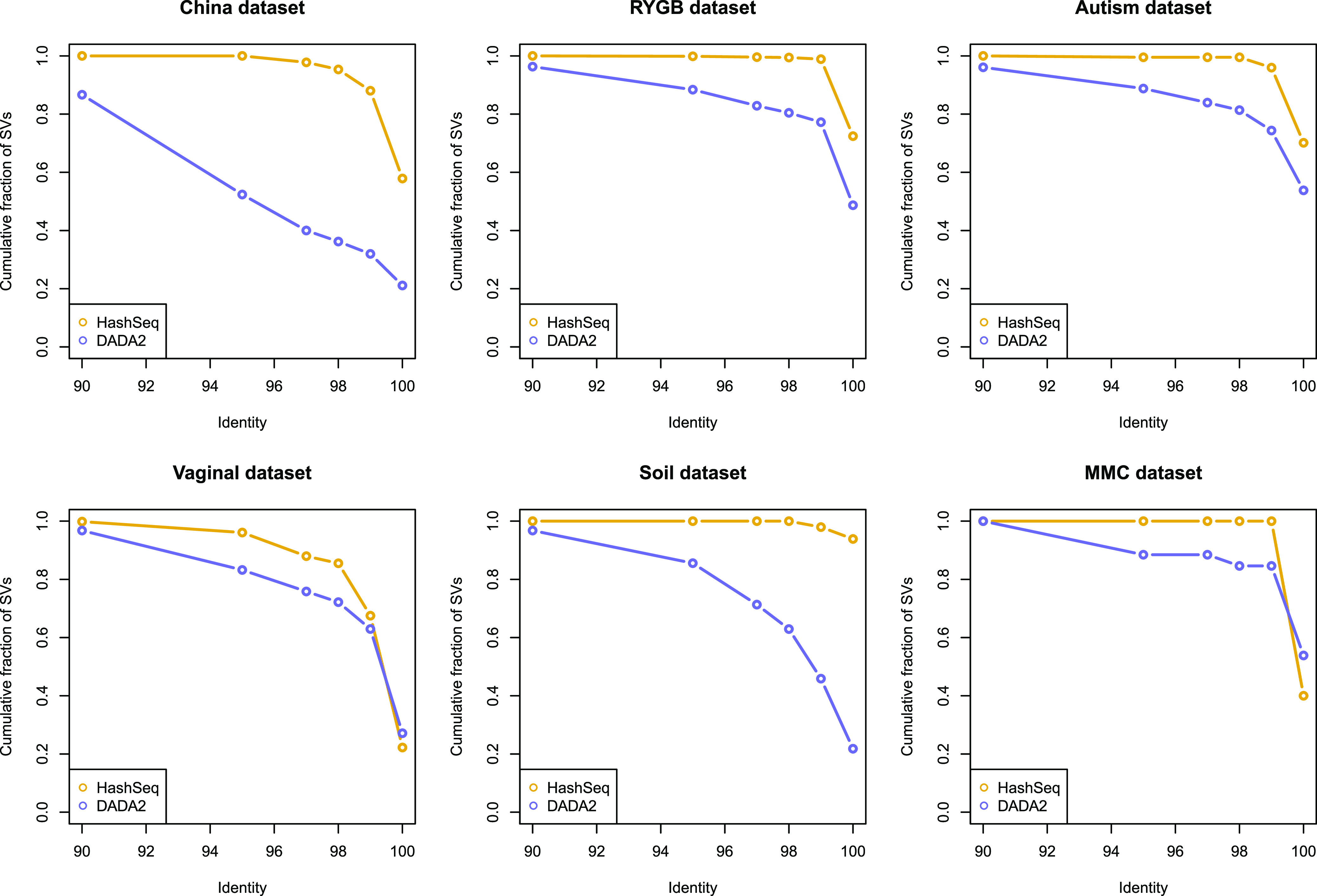
Sequence variants generated by the HashSeq pipeline have a high degree of identity to the SILVA132 database. For each data set, the cumulative fraction of inferred sequence variants for a range of 90 to 100% identity to the SILVA132 database is plotted for both the HashSeq and DADA2 pipelines.

When using a 5% false-discovery rate (FDR), this method results in a considerably lower number of sequence variants than for DADA2 with default parameters for the nonmock biological data sets (Table 1) and for the inference test based on the Poisson distribution described above (Fig. S1). When we mapped the inferred sequence variants with BLAST to the SILVA132 (8, 9) database, the great majority of sequence variants had a high degree of identity (>99%) to the SILVA database (Table 1 and Fig. 6), suggesting that many of the variants that we detected had been previously observed. This supports an assertion that these variants are not sequencing errors. Interestingly, although HashSeq calls more variants in the MMC data set than DADA2, the parent sequences include the eight bacterial taxa that are present in the mock (Listeria monocytogenes, Pseudomonas aeruginosa, Bacillus subtilis, Escherichia coli, Salmonella enterica, Lactobacillus fermentum, Enterococcus faecalis, and Staphylococcus aureus), which further confirms that our clustering strategy is able to find major taxa in a data set. Overall, these results suggest that our normal distribution-based inference approach is often more conservative than DADA2 and less prone to infer spurious variants as true sequences. However, because we do not look for variants in low-abundance regions where the LOESS regression does not show a consistent relationship to sequencing depth, our algorithm is less sensitive to low-abundance true sequences.

**TABLE 1:** Results of mapping of sequence variants inferred by HashSeq and DADA2 to the SILVA132 database using BLAST^*a*^

Data set	Pipeline	No. of SVs	Parents-children	Parents				Children			
				Identity = 100%	100% > identity ≥ 99%	99% > identity ≥ 97%	97% > identity	Identity = 100%	100% > identity ≥ 99%	99% > identity ≥ 97%	97% > identity
China	HashSeq	451	255-196	194	34	21	6	67	102	23	4
	DADA2	4,97		1,050	540	400	2,984				
RYGB	HashSeq	718	402-316	373	21	5	3	147	169	0	0
	DADA2	3,385		1,648	966	191	580				
Autism	HashSeq	422	229-193	214	8	6	1	82	101	9	1
	DADA2	2,398		1,295	493	230	385				
Vaginal	HashSeq	1,305	530-775	222	191	72	45	68	400	195	112
	DADA2	12,106		3,284	4,335	1,562	2,925				
Soil	HashSeq	49	39-10	37	1	1	0	9	1	0	0
	DADA2	5,761		1,256	1,387	1,466	1,652				
MMC	HashSeq	35	9-26	9	0	0	0	5	21	0	0
	DADA2	26		14	8	1	3				

a Shown are the numbers of sequence variants (SVs) inferred by DADA2 and HashSeq as well as the percent identity of the sequence variants to the SILVA132 database.

**FIG 7:**
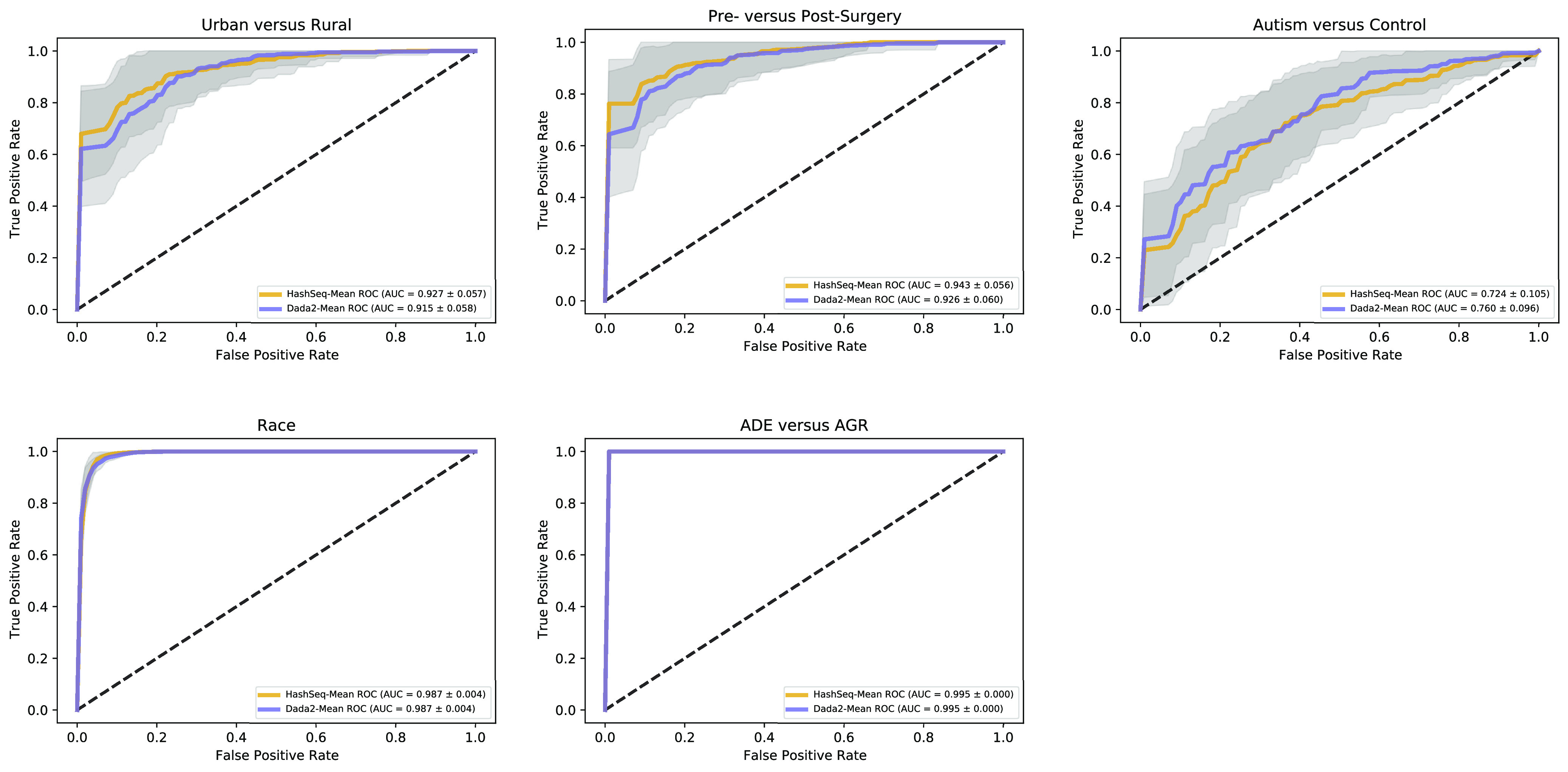
HashSeq performs almost identically to DADA2 in terms of association studies between the gut microbiota and biological variables. Random forest classification was used to study the association between the sequence variants with metadata variable of interest for five different 16S rRNA data sets. The areas under the curves (AUC) of the receiver operating characteristic (ROC) curves were essentially superimposable between our inference-based approach and DADA2. For the China data set, we examined if the gut microbiota can predict rural versus urban samples. For the RYGB data set, we tested if the gut microbiota can predict presurgical versus postsurgical samples. For the vaginal data set, we studied the association between the microbiota and ethnicity (black women versus white women). For the autism data set, we examined if the gut microbiota can predict children with autism versus the control group. For the soil data set, the association between the microbiota and two types of soil, Amazon Dark Earth (ADE) and agricultural soil (AGR), was examined.

Next, for each data set we performed a random forest classification to study the association between the sequence variants with metadata variables of interest in the five publicly available nonmock data sets. Compared to DADA2, our approach performs nearly identically in terms of association studies between the gut microbiota and biological variables (Fig. 7).

**FIG 8:**
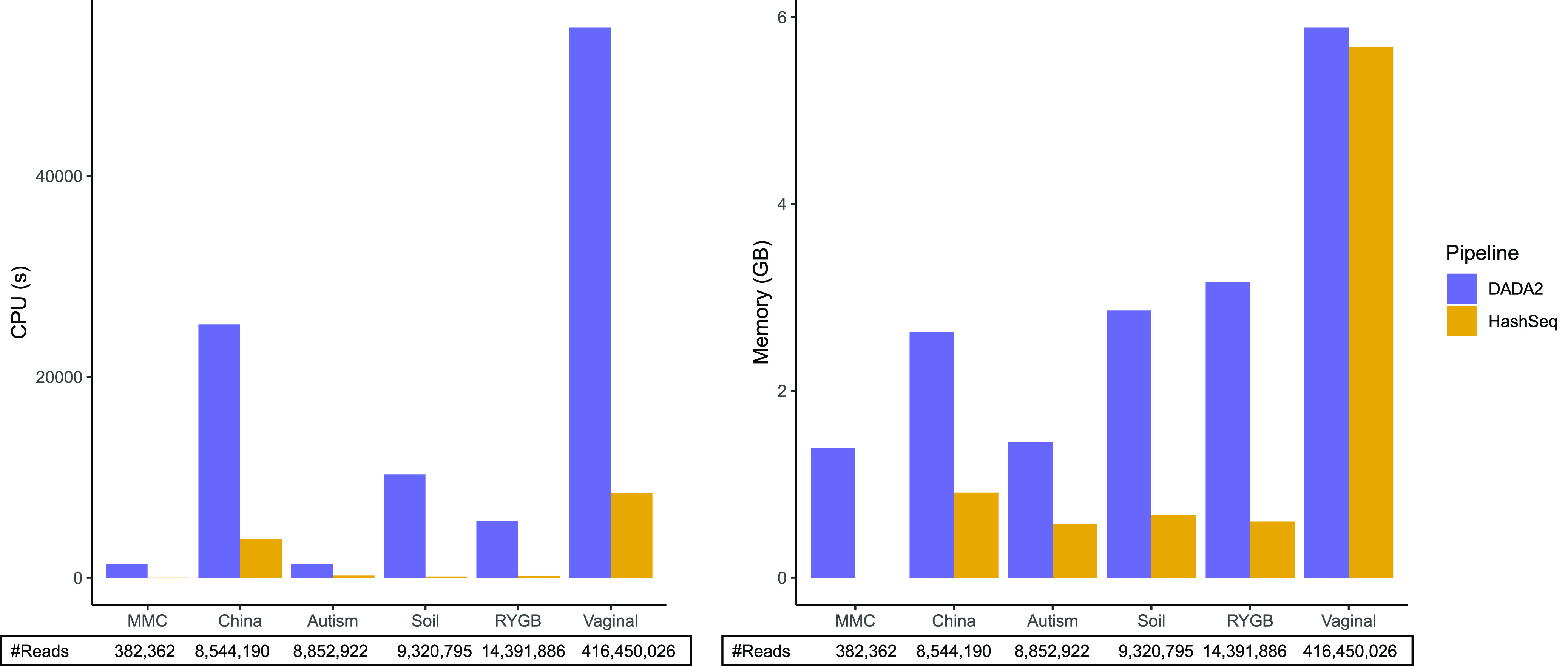
HashSeq is faster and more efficient in memory usage than DADA2. Runtime and memory usage by DADA2 and HashSeq are plotted for each 16S rRNA gene data set.

Finally, we compared runtime and memory usage between our pipeline and DADA2. On average across data sets, our pipeline is 43 times faster than for DADA2 and the memory usage is 3 times less than for DADA2 (Fig. 8). For the vaginal data set, the largest data set, HashSeq was 6.5 times faster than DADA2; however, it was comparable to DADA2 in terms of memory usage.

## DISCUSSION

In this study, we utilized a very simple HashMap-based algorithm to detect all sequence variants in a data set. This resulted unsurprisingly in a large number of one-mismatch sequence variants. We assume that nearly all these spurious sequences were caused by sequencing error. We provide two lines of evidence to support this assertion. First, the number of distinct one-mismatch children for each parent sequence can be well modeled by a simple Poisson process, suggesting that when sequence depth is high enough, every possible one-mismatch variant of a parent sequence can be observed. This seems unlikely to be explained by biological variance. A second line of support for the assertion that most variants are related to sequencing error is the excellent fit to a smooth LOESS curve with sequencing depth over 1,000 sequences (Fig. 5), suggesting that sequencing depth and not the biology of a particular cluster controls the abundances of observed variants.

Given a postulate that nearly all sequence variants are the result of error, a natural approach is to use the background error rate for inference to detect the relatively rare occurrence of a variant that cannot be explained by background sequencing error. This approach of using a background rate to generate *P* values for an event that is reasonably uncommon has long been an approach to inference in genomics (10). A natural question is how to parametrize the expected background rate. Since we know that there is a dependency on sequencing depth, the simplest approach would be a Poisson-based model in which the mean equals the variance. However, the Poisson model failed to control the distribution of one-mismatch children in each cluster, presumably because the Poisson assumption of equal mean and variance was not met. In a similar way, previous studies have shown that when the Poisson distribution is used to test for differential gene expression in RNA-seq data sets, the Poisson-estimated variance is smaller than the observed variance in real data, resulting in increased false discoveries (7, 11). Therefore, overdispersion (where variance is higher than mean) is a general feature of sequence count genomic data sets, including sequence variants, and this problem causes inference based on the Poisson distribution to fail.

We therefore argued that inference of a true variant should utilize a model in which the variance was not constrained to equal the mean. Previous algorithms designed for RNA-seq data sets, such as DESeq and EdgeR, model count data with a negative binomial distribution which assumes that the variance is greater than the mean (7, 12). We preferred the normal distribution over the negative binomial distribution to model the background error for two reasons. First, the negative binomial distribution is not defined when the variance is less than the mean, and although for the majority of sequence variants the variance is greater than the mean, there are still a large number of child sequences that have a mean greater than the variance (Fig. 3). Second, the negative binomial as a count model does not work on log-transformed data, which contain noninteger values. The normal distribution instead gives us more flexibility in terms of the relationship between the mean and variance as well as more naturally allowing for the transformation of count data. Regardless of the limitations of the negative binomial distribution, at high sequencing depth the negative binomial distribution is well approximated by the normal distribution, further justifying the use of the normal distribution.

In order to use a normal distribution-based model to infer true sequences from the background noise, we used the mean and standard deviation predicted by a localized regression fit between mean and standard deviation and parent sequences (Fig. 5). In order to be as conservative as possible, we chose the mean and standard deviation for our inference test to be the higher of the mean and the standard deviation found for each parent directly or predicted by the LOESS regression. This conservative approach detected sequence variants that had a good match to existing variants in the SILVA database, suggesting that many of the variants that we detected had been previously observed and therefore are unlikely to be sequencing errors. This provides further confirmation of the conservative nature of our method.

Our normal-distribution-based algorithm for detection of sequence variants, which we here call HashSeq, has a number of advantages. First, it is very fast and can detect sequence variants in less than 3 h on a single central processing unit (CPU) even for a very large data set. It can run all sequences in a data set together and does not require running sequences from each sample independently. This eliminates any potential problems in which the characteristics of individual samples impact overall variant calling in potentially complex ways. Second, our algorithm, compared to the popular algorithm DADA2, is fairly conservative and calls a lower number of sequence variants as true. The conservative nature of our method potentially increases the power of a study to detect a signal since fewer spurious variants are reported and therefore there will be a lower number of hypothesis to be corrected for in downstream analyses using FDR multiple-hypothesis correction. By determining where the smooth relationship between parent abundance and mean of child abundance breaks down, our algorithm offers a natural way to set a threshold for removing low-abundance variants. This was set at a parent abundance of 1,000 reads for all of our data sets except the largest one, where it was set to 10,000. Setting a low abundance threshold in this way is an appealing alternative to removing taxa based on arbitrary thresholds of rarity or prevalence. In addition, because our algorithm provides explicit *P* values for a null hypothesis that a child sequence was derived from sequencing error of a parent sequence, our results may be easier to interpret than algorithms that do not assign a score to variants or that assign scores based on arbitrary scales.

Finally, our algorithm is simple and is based on a two-parameter model. In contrast, DADA2 assumes that each nucleotide transition has its own parameter which is calculated from the transition probabilities and quality scores. DADA2 assumes that the parameters obtained from quality scores are independent of sequencing depth, while our model explicitly considers background mean and variance as a function of sequencing depth. Despite these differences in parametrization, using our variants or DADA2 variants produces essential identical power for machine learning-based supervised classification.

Our algorithm has some limitations to be noted. First, our algorithm is not sensitive to detect true low-abundance sequences. Therefore, we recommend using more sensitive algorithms, such as DADA2, to detect low-abundance sequence variants. Another limitation is that our algorithm does not consider two or more mismatches. However, we believe that one-mismatch errors are more likely to happen than two or more mismatches, and therefore, the abundances of two or more mismatches will reliably fall below the detection limit of the algorithm (usually <1,000 reads). This assertion is supported by simulation results (Fig. S2) which suggest that variants with more than one mismatch error occur very infrequently in short reads. This assumption of the rarity of multiple-mismatch sequences, however, may not be appropriate for long-read technologies such as PacBio, and this is another potential limitation of our method. Finally, our algorithm does not explicitly model insertions or deletions (indels) and will treat indel events as a separate parent sequence. Users who need to capture indel variation in relationship to a parent might consider use of Deblur or other methods that incorporate multiple-sequence alignments. Our algorithm does not address issues of compositionality and the ways in which compositional artifacts might impact sequence variant calling. Compositionality is an important and appropriate topic for future research.

10.1128/mSystems.00697-21.2 FIG S2 The fraction of all possible unique single mismatches for a sequence can be well fit to a Poisson model as well as simulated data. Black circles in the plot show the relationship between the abundance of parents on a log_10_ scale and the fraction of all possible unique single mismatches that are observed for each parent sequence in the China data set. The red line shows the fraction of all possible single mismatches predicted by a one-parameter Poisson model that includes an error rate of 0.00015. The blue line shows the fraction of all possible single mismatches that are simulated from Java code (see Materials and Methods in the main text) with an error rate of 0.00015 for sequences with different sequence depths. Download FIG S2, PDF file, 0.2 MB. Copyright © 2021 Fouladi et al. 2021 Fouladi et al. https://creativecommons.org/licenses/by/4.0/ This content is distributed under the terms of the Creative Commons Attribution 4.0 International license.

Depending on the goals of the analysis, our algorithm can be run independently or in combination with other sequence variant callers such as DADA2 or Deblur. If the goal of an analysis is to find sequence variants that are associated with a particular experimental condition, our algorithm could be run independently and will produce a series of high-confidence variants which can be fed into statistical models to produce FDR-corrected *P* values. Different thresholds from our algorithm can be used to produce more or fewer variants, which allows users to trade off sensitivity for power in FDR-corrected hypothesis tests of each variant over the threshold. If the desired analysis is machine learning or supervised classification, our results suggest that our algorithm can give results very similar to those of DADA2 with reduced requirements for processing time and memory. A limitation of our algorithm is that it produces only one-off (single-nucleotide polymorphism [SNP]) variants. Combination with other OTU-picking algorithms will be required if users desire a broader clustering approach, with our algorithm used as a preprocessing step to perform initial one-off variant clustering for input into a final OTU-generating algorithm. Likewise, since our algorithm does not provide meaningful inference on low-abundance variants, if low-abundance taxa are of interest to a user, algorithms such as DADA2 are likely more appropriate.

In summary, we have described HashSeq, a very simple and fast algorithm to infer true variants from background sequencing errors. This algorithm can be easily used for small or large 16S rRNA gene data sets generated from a diverse range of ecosystems. Source code is freely available at https://github.com/FarnazFouladi/HashSeq as an R package.

## MATERIALS AND METHODS

### Publicly available data sets.

Six data sets were included in this study: one publicly available microbial mock community (MMC) consisting of three samples (BioProject accession number PRJEB24409) and five publicly available 16S rRNA gene data sets, including three human gut microbiota data sets to which we refer as the China (PRJNA349463; *n* = 80), autism (PRJNA533120; *n* = 81), and Roux-en-Y gastric bypass (RYGB) (Sequence Read Archive [SRA] accession number SRP113514; *n* = 71) data sets, one vaginal microbiota data set (SRP115697; *n* = 2,367), and one soil microbiota data set (PRJEB14409; *n* = 40) (13–18). For all data sets except for the soil data set, forward and reverse reads were merged using the PEAR software (19), and the paired reads were then trimmed to a constant length and shorter reads were discarded (250 nucleotides for the China, vaginal, and MMC data sets, 200 nucleotides for the RYGB data set, and 151 nucleotides for the autism data set). For the soil data set, only forward reads were used, due to concerns about sequence quality for the reverse reads, and the reads were trimmed to 250 nucleotides. For the soil, RYGB, and MMC data sets, primers were present in the public sequences and were removed by our pipeline. Information regarding primers and the variable region of 16S rRNA gene that were sequenced can be found in Table S1. For all data sets, singletons in each sample as well as sequences with N’s were removed.

10.1128/mSystems.00697-21.3 TABLE S1 Data sets used in this study. The table includes the project numbers associated with each data set and the information regarding sequencing, including the sequencing instrument, the variable region in the 16S rRNA gene, and primers where available. Download Table S1, DOCX file, 0.01 MB. Copyright © 2021 Fouladi et al. 2021 Fouladi et al. https://creativecommons.org/licenses/by/4.0/ This content is distributed under the terms of the Creative Commons Attribution 4.0 International license.

### Cluster of sequences composed of a parent and one-mismatch children.

We used a HashMap, a simple data structure, to detect all 16S rRNA gene sequence variants, excluding sequence variants with only one read in a sample (singletons). In our method, sequence variants are sorted according to their abundance. Starting with the most abundant sequence variant (considered a parent sequence), all possible one-mismatch sequence variants in the data set are identified and considered as the one-mismatch children for the parent sequence. Similar searches for parent and child sequences are performed for the remaining sequences until all sequences are assigned as a parent sequence or as a one-mismatch child sequence, resulting in the formation of numerous clusters of sequences that are composed of one parent and one-mismatch children (Fig. 1).

### Poisson model of frequency of one-match child variants.

In order to estimate the rate of observing a one-mismatch sequence variant, we fit our data to a very simple model based on the Poisson distribution. This model has one free parameter which is the probability of a single-nucleotide sequencing error. In this approach, we treat each nucleotide within a set of parent and child sequence variants independently. Given the background error rate of *P* and a parent sequencing depth of P_i_ (a parent belonging to the cluster i), the probability of seeing at least one error for a given nucleotide is given by

(1) $$\text{1}\;-\text{ dpois }\left(0,{\text{ P}}_{\text{i}}\times P/\text{3}\right)$$

“dpois” is an R programming function that calculates density for Poisson distribution. We divide *P* by 3 in the above equation because there are 3 possible distinct nucleotides that can be tabulated as an error (for example, an A base can be erroneously observed as either C, G, or T). If *P* is close to 1, we would expect to always see all three possible variants of the nucleotide, and if *P* is close to zero, we would expect to never see a mutation at that position in the sequence. We argue that we can fit equation 1 to our data sets by considering the fraction of all unique one-mismatch children observed for a parent sequence divided by the number of all possible one-mismatch children as shown in Fig. 1 and described in Results. If *P* is high, then we would expect to see most of all the possible one-mismatch variants of a parent sequence, and if *P* is low, we would expect to see few.

This model makes a number of simplifying assumptions. A key assumption is that the error rate *P* can be estimated independently for each nucleotide and that not considering sequences with more than one nucleotide difference between parent and children does not bias our error rate estimate. We think this is a reasonable assumption, as simulating a polymerase with the same error rate as the Poisson equation above and examining the resulting distribution of one-mismatch children observed from among all resulting sequences yields an essentially identical distribution as the Poisson equation above (the simulation code is available here at https://github.com/afodor/metagenomicsTools/blob/master/src/binomFit/HowManyVariants.java; see also Fig. S2). This concordance occurs because the overall error rate is low enough that sequences with more than one mismatch occur infrequently and can therefore be ignored without altering our baseline error rate estimate. For example, for a 250-bp sequence length with a *P *value of 0.00015 for error rate, sequences with more than one mismatch are seen only in about 1 of 1,600 sequences in our simulation code. Obviously, this assumption of independence of sequence variants that allows us to ignore sequences with multiple mismatches becomes more problematic for read lengths greater than the 250 bp that we examined in this study and for overall higher error rates.

In addition, in order to see whether the estimated error rate derived from the presence or absence of one-mismatch variants using the above model can be used to predict the background abundance of sequence errors and therefore to infer true variants whose abundances are above the background noise, we used a Poisson test using “poisson.test” in R with the following parameters:

(2) $$\text{Poisson}.\text{test }(C_{\text{ji}},{\text{P}}_{\text{i}},\;P/\text{3},\text{ alternative}=“\text{greater}”)$$

where *C*_ji_ is the abundance of a jth child sequence of the cluster i, P_i_ is the abundance of a parent sequence of the cluster i, and *P* is the estimated error rate from the Poisson model above (equation 1). *P* values generated by the Poisson test (Fig. S1) were adjusted for multiple-hypothesis testing using the Benjamini-Hochberg procedure.

### Normal distribution of the background noise.

As equation 2 based on the Poisson distribution underestimates the abundances of one-mismatch children (see Results), we further examined if the abundances of one-mismatch children can be better fit by the normal distribution. For this purpose, abundances of sequence variants were log_10_ transformed and their histograms were plotted for each parent sequence. Next, the mean and standard deviation for each parent were calculated. The relationship between the mean abundance of children and the parent sequences as well as the standard deviation abundance of children and the parent sequences were fitted to a local regression, or locally estimated scatterplot smoothing (LOESS). We show that for parent sequences with depths above 1,000 reads, the LOESS regression is a reasonable fit for most data sets; however, for parents with depths below 1,000 reads, variance of the means and standard deviations is increased due to the sparsity of one-mismatch children, and therefore, the LOESS regression does not fit as well (Fig. 5). Therefore, as a default, sequence variants with total abundance of less than 1,000 across all samples are filtered (i.e., removed) in our pipeline. This threshold of 1,000 can be changed by users based on their data. For example, for the vaginal data set, we increased the threshold to 10,000, as the sequencing depth is significantly higher for this data set than for other data sets and the LOESS regression is a good fit when sequences have depths higher than 10,000 reads (Fig. 5).

The means and standard deviations estimated from the LOESS regression were assumed to be the background noise, and therefore, any variant above this background noise would be called a true sequence variant. Based on this assumption, for each child variant, a one-sided *P* value was generated using the “pnom” function in R and the following formula:

(3) $${\text{pnorm[log}}_{10}(C_{\text{ji}}),\text{ lower}.\text{tail }=\text{ FALSE},\;M_{\text{i}},\;S_{\text{i}}]$$

(4) $$M_{\text{i}}=\text{max }\left[\text{mean }\left(C_{\text{i}}\right),\text{ mean for LOESS fit}\right]$$

(5) $$S_{\text{i}}=\text{maximum }\left[\text{SD }\left(C_{\text{i}}\right),\text{ SD for LOESS fit}\right]$$

where *C*_ji_ is the abundance of jth child of cluster i and *M*_i_ and *S*_i_ are, respectively, the estimated mean and standard deviation (SD) of cluster i, which are the maximum of the mean and standard deviation predicted by the LOESS regression from the abundance of the parent sequence in cluster i and the mean and standard deviation estimated directly from the child abundance of cluster i (equations 4 and 5). Taking the maximum of the mean and standard deviation enables us to be more conservative, especially for low-abundance sequences where data become sparse and the LOESS fit is less reliable. *P* values generated by the “pnorm” test were adjusted for multiple-hypothesis testing using the Benjamini-Hochberg procedure. Corrected *P* values of less than 0.05 were considered significant, rejecting the null hypothesis that the variant child is a sequence error.

### Comparison to DADA2.

We compared the performance of HashSeq to the performance of the DADA2 pipeline (5). For this purpose, reads for each data set were trimmed to the same length as discussed above. Trimming was performed with the function “filterAndTrim” in DADA2 with default parameters. Inference of sequence variants was performed as described in https://benjjneb.github.io/dada2/bigdata.html with default parameters. Filtering and inference were performed using separate scripts in order to better compare the run time and memory usage between the DADA2 algorithm and HashSeq.

In order to compare DADA2 and our algorithm, we used “blastn” to map sequence variants inferred from both algorithms to the SILVA132 database. Percent identity was calculated as

(6) [alignment length – (number of mismatches + gaps)]/maximum (alignment length, sequence length)×100

where “sequence length” is the known length of the query sequence and all other parameters were reported by BLAST. This formula penalizes both mismatches and gaps in either the sequence or the alignment. For each sequence variant, the first hit with the highest bit score with the database was selected.

Finally, we compared DADA2 and our algorithm in terms of associations between the inferred sequence variants and the variable of interest in the metadata. For this purpose, we performed random forest classification for each algorithm and data set with four cross-validations and 10 repeats using RandomForestClassifier with 100 decision trees and RepeatedKFold methods from the Scikit-learn library in python 3.8.1.

### Data availability.

Our pipeline is written in Java (JDK 1.8) and R (4.0.2) but can be installed as an R package and run from an R environment. Source code with instructions for installing HashSeq package can be found at https://github.com/FarnazFouladi/HashSeq. All codes and figures for the analyses of this manuscript can be found at https://github.com/FarnazFouladi/HashSeq_Manuscript.
