# Supplementary figures and images for: HashSeq: a Simple, Scalable, and Conservative De Novo Variant Caller for 16S rRNA Gene Data Sets
Source: mSystems. 2021 Nov 9;6(6):e00697-21. doi: 10.1128/mSystems.00697-21 (PMC8577285; doi:10.1128/mSystems.00697-21)

**China**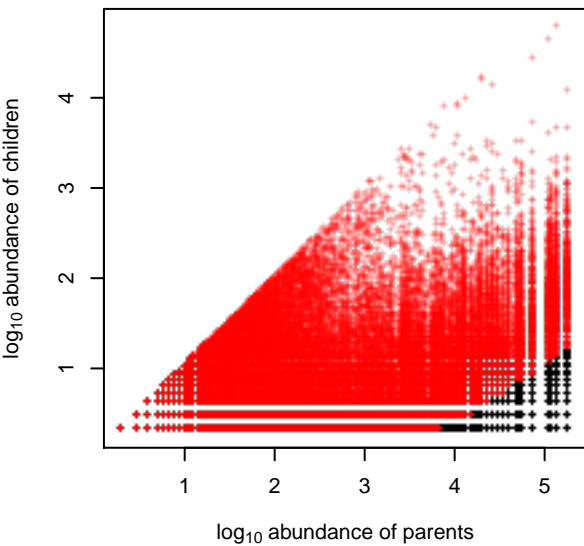**RYGB**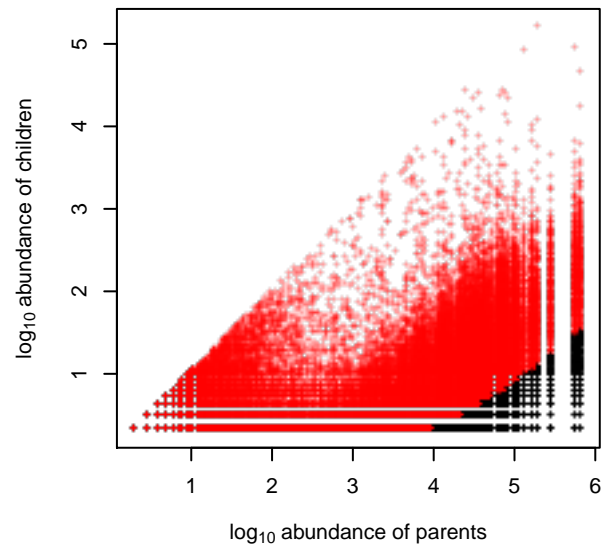**Autism**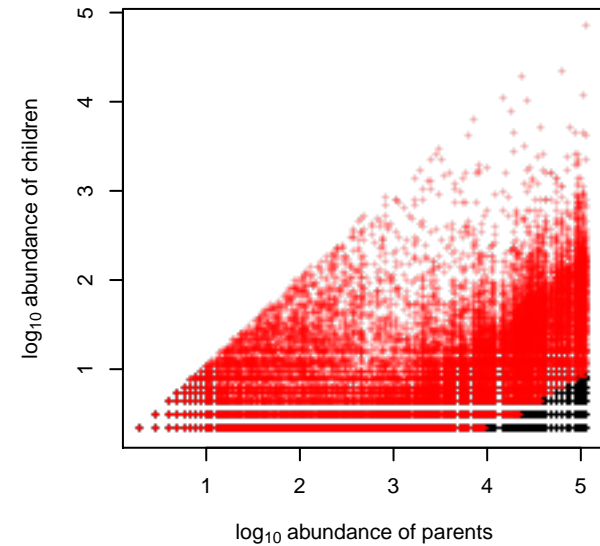**Vaginal**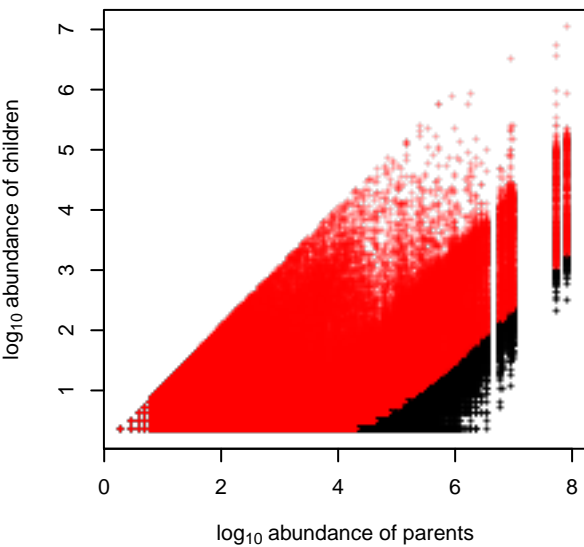**Soil**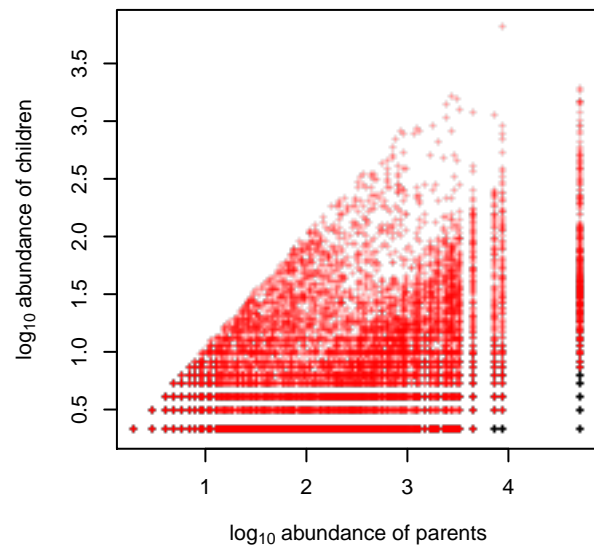**MMC**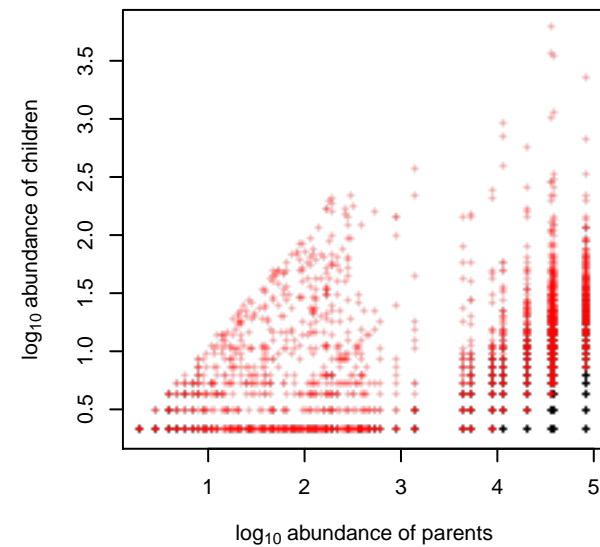

Supplement: FIG S1 [file msystems.00697-21-sf001.pdf]

**Error rate = 0.00015**

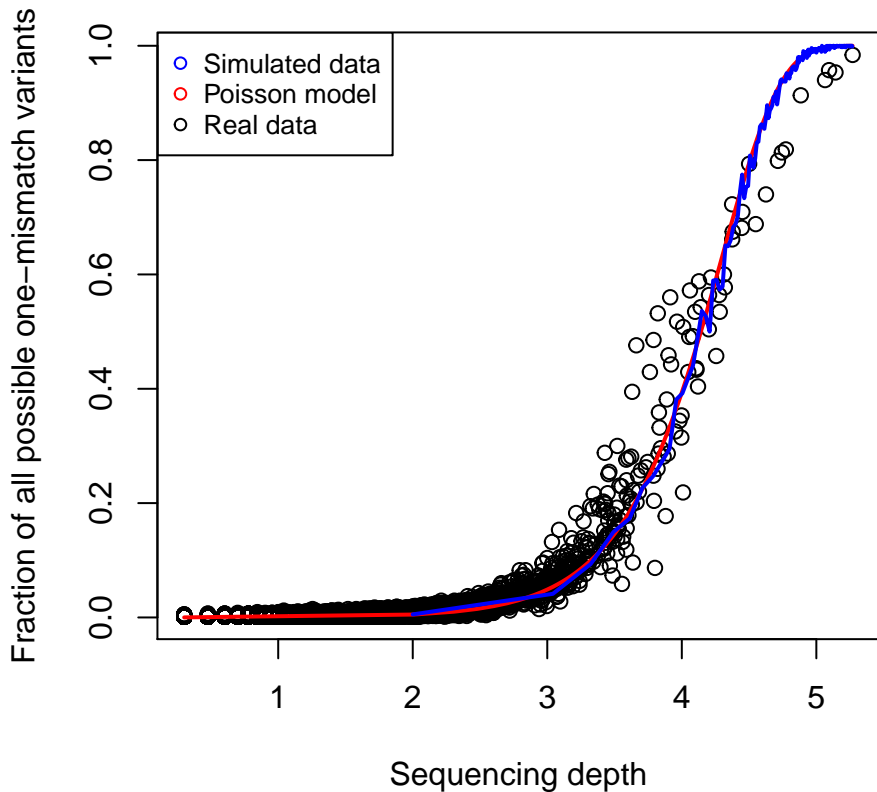

Supplement: FIG S2 [file msystems.00697-21-sf002.pdf]
