# Supplementary material for: HashSeq: a Simple, Scalable, and Conservative De Novo Variant Caller for 16S rRNA Gene Data Sets
Source: mSystems. 2021 Nov 9;6(6):e00697-21. doi: 10.1128/mSystems.00697-21 (PMC8577285; doi:10.1128/mSystems.00697-21)
Supplement: TABLE S1 [file msystems.00697-21-st001.docx]

| **Datasets** | **Project#** |  | **Region** | **Forward primer** | **Reverse primer** |
| --- | --- | --- | --- | --- | --- |
| autism | PRJNA533120 | MiSeq | V4 | GTGCCAGCMGCCGCGGTAA | GGACTACHVGGGTWTCTAAT |
| RYGB | SRP113514 | MiSeq | V4 | TCGTCGGCAGCCAGTGATGTGTATAAGAGACAGGTGCCAGCMGCCGCGGTAA | GTCTCGTGGGCTCGGAGATGTGTATAAGAGACAGGGACTACHVGGGTWTCTAAT |
| China | PRJNA349463 | MiSeq | V4 | Not available | Not available |
| Soil | PRJEB14409 | MiSeq | V3-V4 | CCTACGGGNGGCWGCAG | GACTACHVGGGTATCTAATCC |
| vaginal | SRP115697 | HiSeq 2500 | V4 | Not available | Not available |
| MMC | PRJEB24409 | MiSeq | V4-V5 | CAGCMGCCGCGGTAATACG | Not available |

**Supplementary Table 1. Datasets used in this study.**

The table includes the project numbers associated with each dataset and the information regarding sequencing, including the sequencing instrument, the variable region in the 16S rRNA gene, and primers where available.
